# Restless abdomen: a spectrum or a phenotype variant of restless legs syndrome?

**DOI:** 10.1186/s12883-020-01875-1

**Published:** 2020-08-12

**Authors:** Xi-Xi Wang, Xiao-Ying Zhu, Zan Wang, Jian-Wei Dong, William G. Ondo, Yun-Cheng Wu

**Affiliations:** 1 Department of Neurology, Shanghai General Hospital, Shanghai Jiao Tong University School of Medicine, Shanghai, 200080 P.R. China; 2 Shanghai General Hospital of Nanjing Medical University, Nanjing, 210029 P.R. China; 3 Department of Neurology, The First Hospital of Jilin University, 71 Xinmin Street, Changchun, Jilin, 130021 P.R. China; 4 Department of Neurology, Changchun Central Hospital, Changchun, P.R. China; 5 Department of Neurology, Methodist Hospital, Weill Cornell Medical School, Houston, TX USA

**Keywords:** Restless abdomen, Restless legs syndrome, Insomnia, Dopaminergic therapy

## Abstract

**Background:**

With the growing awareness of restless legs syndrome (RLS), sensory disorders similar to RLS but initially confined to the arms, abdomen, and perineum have been reported. One of them is restless abdomen, which refers to a restless sensation in abdomen. Our study is designed to evaluate the clinical phenotype of restless abdomen and investigate its relationship with RLS.

**Methods:**

We enrolled 10 patients with restless abdomen according to RLS diagnostic criteria, excluding the requiring of leg involvement. Laboratory examinations were performed to exclude mimics and notable comorbidities.

**Results:**

All 10 patients had RLS like symptoms in the abdomen and otherwise satisfied all other RLS diagnostic criteria, and responded to dopaminergic therapy.

**Conclusions:**

Neurologists and gastroenterologists should be aware that RLS-related restlessness can occur in extra-leg anatomy in the absence of episodes of worsening or augmentation of restlessness.

## Background

Restless legs syndrome (RLS) is a common neurologic sleep related movement disorder, characterized by unpleasant sensations localized deep in the lower limbs associated with an urge to move the legs, which worsens at rest, and in the evening or night hours [1–3]*.*

As awareness of RLS has grown, several small reports describe sensory disorders similar to RLS initially confined to the arms, abdomen, and perineum [4–10]. These reported cases also partially meet the diagnostic criteria of RLS. Restless abdomen, which has been referred to some case reports, means a restless sensation in the abdomen [11, 12]. It meets all other criteria except anatomy and has been reported in several case studies (Table 1).

**Table 1:** Features of ever reported restless abdomen cases

	Age & Gender	Medical history	Duration of symptoms, y	Main clinical manifestations	Diagnostic criteria					Supportive criteria					Nature of the disease
					D1	D2	D3	D4	D5	S1	S2	S3	S4	S5	
2003 Italy	72 M	Facial tics since adolescence	4	An unpleasant restless sensation originating in his lower abdomen, spreading then to the lower limbs and associated with a compelling need to move the legs.	Y	Y	Y	Y	Y	Y	Y	N	/	/	Part of the phenotypic spectrum of RLS
2011 Spain	62 M	/	14	Nocturnal awakenings due to a “nervous twitchy feeling” in his abdomen.	Y	Y	Y	Y	Y	N	Y	N	Y	Y	A phenotype variant of RLS
	62 M	/	6	An unpleasant “abnormal tickling” in his abdomen	Y	Y	Y	Y	Y	N	Y	N	Y	Y	
	62 F	/	1	Electric currents, shocks, or sparks deep in her belly	Y	Y	Y	Y	Y	N	Y	N	Y	Y	
2014 Japan	43 F	/	0.17	Uncomfortable sensations including ‘twitching’ in his abdomen	Y	Y	Y	Y	Y	N	Y	N	Y	Y	‘Periodic abdominal movements’.
2015 Italy	81 M	HBP, HLP, AAA, CAD, mild renal insufficiency and left renal artery stenosis.	34	Abdominal ‘cramps’ associated with unpleasant restless sensation of discomfort with the urge to move occurring in the evening when lying in relaxed wakefulness and when trying to fall asleep.	Y	Y	Y	Y	Y	N	Y	Y	Y	N	A variant of periodic limb movement in wakefulness (LM/PLMW)

*M* male, *F* female

D1: the urge to move/unpleasant sensation, D2: at rest, D3: night, D4: activity, D5: Exclude other, such as drugs and peripheral neuropathy

S1: Legs involved or not, S2: Insomnia, S3: family history, S4: Responsive to dopaminergic therapy, S5: PLMS in sleep, Y: yes, N: not

*HBP* high blood pressure, *HLP* hyperlipidemia, *AAA* abdominal aortic aneurism, *CAD* coronary artery disease

We collected 10 patients in whom RLS symptoms existed in the abdomen but satisfied all other RLS diagnostic criteria.

## Methods

### Patients

This was a retrospective study. Patients were enrolled from three centers: Shanghai General Hospital, The First Hospital of Jilin University and Changchun Central Hospital. Ten patients with a diagnosis of RLS and abdominal involvement, obtained from a total of 665 RLS patients, were enrolled from Jan 2014 to Dec 2019. The diagnosis of RLS was made according to the 2014 International Restless Legs Syndrome Study Group (IRLSSG) diagnostic criteria [2]. The criteria include: 1) an urge to move the legs, which is usually but not always accompanied by uncomfortable and unpleasant sensations in the legs; 2) urge to move the legs and uncomfortable sensations begin or worsen during periods of rest or inactivity such as lying down or sitting; 3) urge to move and uncomfortable feelings are partially or totally relieved by movement, at least as long as the activity continues; 4) the urge to move the legs and any accompanying unpleasant sensations during rest or inactivity only occur or are worse in the evening or night than during the day; 5) the occurrence of the above features is not solely accounted for as symptoms primary to another medical or behavioral condition. The study was approved by the ethics committee of the Shanghai General Hospital Institutional Review Board, and The First Hospital of Jilin University and Changchun Central Hospital accepted that IRB approval documents because this study is a retrospective study without any interventions.

### Demographics and symptoms

Patients’ home address, age, gender, education, occupation, marital status, smoking history, alcohol history, coffee and tea history, height, weight and Body Mass Index (BMI) were all collected. The main symptoms and characteristics of RLS were also recorded.

### Laboratory examination

Laboratory assessments included ferritin, transferrin, serum iron, total iron binding capacity (TIBC), hemoglobin (Hb), erythropoietin (EPO), routine biochemistry test and transferrin saturation (TSAT). TSAT is calculated by the ratio of serum iron to TIBC.

### Clinical evaluation

Severity of RLS was evaluated by the International RLS Rating Scale (IRLSRS). Individual anxiety and depression states were assessed by Hamilton Anxiety Scale (HAM-A) and Hamilton Depression Scale (HAM-D) respectively.

### Statistical analysis

SPSS software was used for statistical analyses. Quantitative data with a normal distribution were presented as “mean ± standard deviation (SD)”. Probability (P) values<0.05 were considered significant.

## Results

Detailed demographic information and clinical features of 10 patients with restless abdomen are shown in Tables 2 and 3. These patients presented to our clinics because of insomnia and abnormal feelings in their abdomen. One patient (case 7) reported that she had experienced a hysteromyomectomy before and the other nine patients denied any gastrointestinal disorders or abdominal surgical history.

**Table 2:** Demographic information of RLS patients with abdomen involvement

Demographics	Case 1	Case2	Case3	Case4	Case5	Case 6	Case 7	Case 8	Case 9	Case 10
**BMI (kg/m2)**	24.97	22.37	32.89	23.45	18.67	15.80	22.85	24.77	23.44	25.25
**Marital status**	Married	Married	Married	Married	Married	Married	Married	Married	Married	Married
**Age at onset, y**	48	36	62	9	27	44	71	69	72	75
**Symptom duration, y**	18	26	4	50	38	20	0.83	1	14	3
**Smoking history**	N	N	Y	N	N	N	N	N	N	Y
**Alcohol, coffee or tea**	N	N	N	N	N	N	N	N	N	N
**Ethnic Han**	Y	Y	Y	Y	Y	Y	Y	Y	Y	Y
**Home address**	Central China	East China	East China	Northeast China	Central China	East China	Northeast China	Northeast China	Northeast China	Northeast China
**Occupation**	Farmer	Retiree	Retiree	Kindergarten teacher	Farmer	Retiree	Retiree	Retiree	Retiree	Retiree

*BMI* body mass index, *N* no; *Y* yes

**Table 3:** Clinical features of RLS patients with abdomen involvement

	Case 1	Case 2	Case 3	Case 4	Case 5	Case 6	Case 7	Case 8	Case 9	Case 10
**Associated features**										
Family history	–	–	–	+	–	–	–	–	–	–
Response to dopaminergic therapy	+	+	+	+	+	+	+	+	+	+
IRLSRS (scores)	Severe(21)	Severe (21)	Severe (29)	Very severe (35)	Severe (27)	Moderate (20)	Very severe(31)	Very severe(32)	Severe (29)	Very severe(32)
Classification	Chronic-persistent	Chronic-persistent	Chronic-persistent	Intermittent	Chronic-persistent	Chronic-persistent	Chronic-persistent	Chronic-persistent	Chronic-persistent	Chronic-persistent
RLS symptom fluctuation with seasonal trends	Worse in cold weather	Worse in wet weather	U	Worse in summer	U	U	U	U	U	U
Distribution of the sensations	Front of thigh and abdomen	Legs, arms and abdomen	Upper thigh, genital area and abdomen	Legs, arms, hip and abdomen	Legs and abdomen	Knee, popliteal fossa, calf and abdomen	Abdomen	Abdomen	Chest and abdomen	Legs and abdomen
Type of sensations	Itching	Crawling	Soreness, formication and itching	Indescribable discomfort	Indescribable discomfort	Itching and soreness	Soreness	Indescribable discomfort	Itching	Numbness
**HAMA (scores)**	Possible anxiety(9)	Without anxiety(5)	Obvious anxiety(23)	Obvious anxiety(25)	Possible anxiety(9)	Exist anxiety(15)	Possible anxiety(12)	Exist anxiety(20)	Without anxiety(5)	Obvious anxiety(21)
**HAMD (scores)**	Possible depression(13)	Possible depression(11)	Exist depression(17)	Exist depression(18)	Possible depression(12)	Exist depression(17)	Exist depression(18)	Possible depression(12)	Without depression(4)	Exist depression(14)
**Therapy**	Pramipexol 0.25 mg/n	Pramipexol 0.25 mg/n	Pramipexol 0.25 mg/n and antidepressant	Pramipexol 0.125 mg/n	Pramipexol 0.25 mg/n	Pramipexol 0.25 mg/n	Pramipexol 0.25 mg/n and gabapentin 0.3 g/d	Pramipexol 0.25 mg/n and gabapentin 0.3 g/d	Pramipexol 0.25 mg/n and gabapentin 0.3 g/d	Madopar 125 mg/d

*U* unrelated, *HAMA* Hamilton Anxiety Scale, *HAMD* Hamilton Depression Scale

Cases (8 females and 2 males), aged 68.8 ± 7.67 years (range, 59–86 years), were collected from Jan 2014 to Dec 2019, at Shanghai General Hospital, the First Hospital of Jilin University and Changchun Central Hospital. They were all outpatients. Only one patient (case 4) described that her grandmother, mother and daughter had the similar regular RLS symptoms in their legs, while the other 9 cases (90%) denied any family history of RLS. Two patients (case 2, 10) had a history of hypertension, 2 patients had a history of diabetes. One patient (case 3) reported she had a history of anemia 20 years ago. They all denied the history of end-stage renal failure, sleep apnea syndrome, Parkinson’s disease, essential tremor, or demyelinating disorders.

**Fig. 1:**
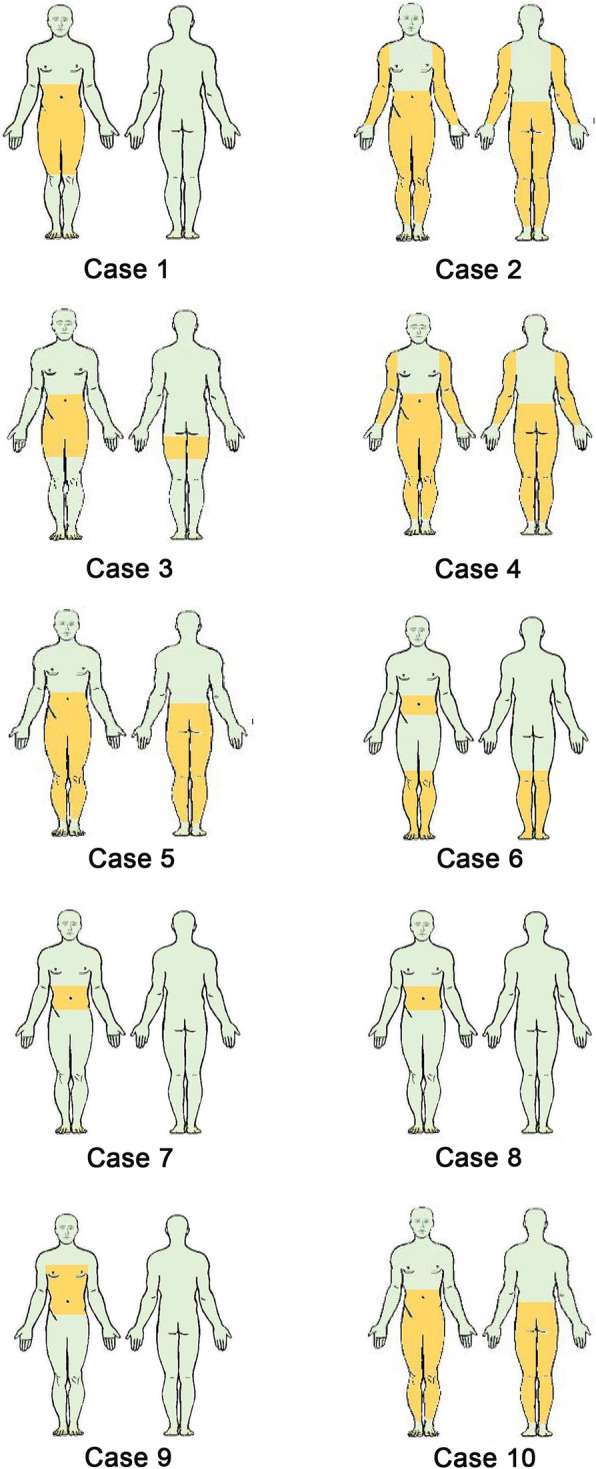
An overview of distribution the restlessness of 10 patients. The areas painted yellow are where the patient has unpleasant symptoms. Case 1: Front of thigh and abdomen. Case 2: Legs, arms and abdomen. Case 3: Upper thigh, genital area and abdomen. Case 4: Legs, arms, hip and abdomen. Case 5: Legs and abdomen. Case 6: Knee, popliteal fossa, calf and abdomen. Case 7: Abdomen. Case 8: Abdomen. Case 9: Chest and abdomen. Case 10: Legs and abdomen

The age at symptom onset was 51.3 ± 21.18 years and the duration were 17.48 ± 15.84 years (ranged from 10 months to 50 years). The distribution of restlessness in 10 patients are shown in Fig. 1. Two patients reported symptoms confined to the abdomen, one had abdomen and chest anatomy, five had abdomen and legs anatomy, and 2 had abdomen, legs and arms anatomy. The type of sensations mainly included itching, crawling and formication, numbness, soreness and indescribable discomfort. In those 7 with limb involvement, the subjective symptoms were similar in the limb and abdomen. Patients’ discomfort occurred or worsened at night/late evening and at rest, which could be partially improved immediately after activity. However, the symptoms appeared again when the patients lay down. Three patients reported that their restless abdomen symptoms fluctuated with seasonal trends or weather variation, among whom 1/3 (33.33%) had worsening symptoms in summer, 1/3 (33.33%) in cold weather and 1/3 (33.33%) in wet weather. The other 7 patients didn’t notice or couldn’t remember whether their symptoms were related with seasonal fluctuation. One case was intermittent disease course while the other 9 had a chronic-persistent disease course.

Nine of the 10 non-anemic restless abdomen patients underwent evaluation of iron parameters. The average ferritin was 90.43 ± 35.65 μg/L, serum iron 14.4 ± 3.95 umol/L, TIBC 60.24 ± 11.15 umol/L, Hb 124.41 ± 6.22 g/L, EPO 15.01 ± 4.77 umol/L, TSAT 24.88 ± 8.10%. According to the defined low iron status, serum ferritin<75 μg/L or serum ferritin ≧75 μg/L but TSAT<20% with normal Hb level, 5 patients (50%) were considered as low iron status. Even excluding 2 patients (case 5, 9) with clear iron deficiency found from the single perspective of serum iron, there were still 3 patients with low iron status. Other routine biochemistry tests were normal.

The mean IRLSRS score at the first-visit was 27.7 ± 5.04, HAM-A was 14.4 ± 7.09, HAM-D was 13.6 ± 4.08.

Video-polysomnography performed in 3 subjects revealed a mean PLMS index of 23.5/h in case 7, 38.2/h in case 8 and 18.7/h in case 9.

## Discussion

We describe 10 cases from three centers with symptoms involving the abdomen, 2 of which were isolated to the abdomen, 1 with additional chest involved and 7 of which included legs. The technical definition of RLS requires leg involvement but does not exclude concurrent extra-leg involvement, therefore our cases with isolated abdominal involvement do not meet criteria for RLS, yet responded similarly to treatment, suggesting similar pathophysiology. Abdomen involvement in RLS is not common. In our previous single center study, we included 359 primary RLS patients and found a total of 61 (17%) patients had extra body parts involvement beyond the legs, only 5 patients (1.4%) of which presented abdomen involvement and are included in this study [13]. There are 10 (1.5%) restless abdomen cases were found among whole 665 RLS patients from this study.

Previous studies have revealed that there is female gender preponderance in RLS patients. In our Chinese population, we also found a female:male ratio of approximately 2:1 [13, 14]. However, cases of restless abdomen reported so far have been mostly male [12, 15, 16]. In contrast, our study found only two male patients (20%). A number of studies have found that the higher prevalence of RLS in women may be relevant to hormonal changes in the body [17–19]. However, to the best of our knowledge, no one has studied the sex distribution of restless abdomen. Whether a gender bias exists in cases of restless abdomen remains unclear. The difference between our reported cases and those previously reported restless abdomen may be due to 1) the restless abdomen cases don’t have a clear gender preference as a matter of fact. 2) Racial/ethnic differences between previously reported cases and cases herein. 3) Random chance, given the small sample size.

Compared to our previous study in the general RLS population [13], restless abdomen cases had a higher age at symptom onset (51.3 ± 21.18 vs. 38.0 ± 15.3 years). Since early onset of RLS correlates with the likelihood of a positive family history of RLS, the relatively older age at onset in abdominal RLS compared to the general RLS population is consistent with this result, suggesting more influence of non-genetic causes.

All the patients we reported here had abnormal sensation that led to poor sleep. This feature is consistent with several previously reported cases. These patients always suffered from severe insomnia but may have sought care from a gastroenterologist rather than neurologist or sleep specialist [12, 20]. Therefore, it is important for neurologists, especially those who work in sleep clinics, to be able to identify patients with restless abdomen.

Augmentation can increase anatomy of RLS involvement, most commonly to the arm. Augmentation was not likely culpable in these patients, because only 2 of our subjects were on dopaminergics at onset of abdominal symptoms, for 4 and 3 years, and neither demonstrated other evidence of augmentation (Table 3).

Restless abdomen can be considered as a part of the phenotypic spectrum of RLS or a separate variant of RLS. Seven of our cases had discomfort in abdomen accompanied with legs discomfort and satisfied the diagnostic criteria for RLS. These characteristics support that cases are a part of the phenotypic spectrum of RLS. However, compared to RLS in our general population, this population was older at onset, lacked a family history of RLS suggesting a phenotypic variant of RLS. Therefore, considering that distribution of the restlessness of 8 of our cases is not restricted to the abdomen and these patients with abdominal symptoms responded to dopaminergics, we can’t rule out the possibility that these 8 cases here are a part of the phenotypic spectrum of RLS.

There are some limitations in our research. Firstly, it has been reported in the past literature that such patients have a high incidence of PLMS [21], but we did not ask about this in detail when collecting the history. Furthermore, not all patients received PSG examinations. Secondly, there is a lack of follow-up data such as IRLSRS, HAMD, and HAMA and so on. Nevertheless, we feel this report should add to recognition of an unusual phenotype which can be successfully treated after correct diagnosis.

## Conclusions

In conclusion, neurologists and gastroenterologists should be aware that RLS-related restlessness can occur in other parts of the body in the absence of episodes of worsening or augmentation of restlessness. Severe insomnia is a good key to identifying such patients. Since dopaminergic therapy significantly improves sleep problems and quality of life in patients, the diagnosis of the variant of RLS—restless abdomen is of great importance.

## Supplementary information

**Additional file 1.** Supplementary file: Raw data of these patients enrolled in our study.

## Data Availability

All data generated or analyzed during this study are included in its supplementary information files.

## Abbreviations

RLS: Restless legs syndrome
IRLSSG: International Restless Legs Syndrome Study Group
BMI: Body Mass Index
TIBC: Total iron binding capacity
Hb: Hemoglobin
EPO: Erythropoietin
TSAT: Transferrin saturation
IRLS-RS: International RLS Rating Scale
HAMA: Hamilton Anxiety Scale
HAMD: Hamilton Depression Scale
SD: Standard deviation
P: Probability
EOR: Early onset RLS

## References

[1] Muth CC (2017). Restless Legs Syndrome. JAMA.

[2] Allen RP, Picchietti DL, Garcia-Borreguero D, Ondo WG, Walters AS, Winkelman JW (2014). Restless legs syndrome/Willis-Ekbom disease diagnostic criteria: updated international restless legs syndrome study group (IRLSSG) consensus criteria--history, rationale, description, and significance. Sleep Med.

[3] Winkelman JW, Armstrong MJ, Allen RP, Chaudhuri KR, Ondo W, Trenkwalder C (2016). Practice guideline summary: treatment of restless legs syndrome in adults: report of the guideline development, dissemination, and implementation Subcommittee of the American Academy of neurology. Neurology.

[4] Horvath J, Landis T, Burkhard PR (2008). Restless arms. Lancet.

[5] Wylie K, Levin R, Hallam-Jones R, Goddard A (2006). Sleep exacerbation of persistent sexual arousal syndrome in a postmenopausal woman. J Sex Med.

[6] Umehara H, Sumitani S, Ohmori T (2010). Restless legs syndrome with chest and back restlessness as the initial symptom. Psychiatry Clin Neurosci.

[7] Aquino CC, Mestre T, Lang AE (2014). Restless genital syndrome in Parkinson disease. JAMA Neurol.

[8] Suzuki K, Miyamoto M, Uchiyama T, Miyamoto T, Matsubara T, Hirata K (2016). Restless bladder in an elderly woman: an unusual feature or a variant of restless legs syndrome?. Intern Med.

[9] Michaud M, Chabli A, Lavigne G, Montplaisir J (2000). Arm restlessness in patients with restless legs syndrome. Mov Disord.

[10] Freedom T, Merchut MP (2003). Arm restlessness as the initial symptom in restless legs syndrome. Arch Neurol.

[11] Esaki Y, Kitajima T, Tsuchiya A, Hirose M, Torii Y, Fujita S (2014). Periodic abdominal movements. Psychiatr Clin Neurosci.

[12] Perez-Diaz H, Iranzo A, Rye DB, Santamaria J (2011). Restless abdomen: a phenotypic variant of restless legs syndrome. Neurology..

[13] Zhu XY, Wu TT, Wang HM, Ni LY, Li X, Liu Y (2019). Clinical features and subtypes of restless legs syndrome in Chinese population: a study of 359 patients. Sleep Med.

[14] Gao X, Schwarzschild MA, Wang H, Ascherio A (2009). Obesity and restless legs syndrome in men and women. Neurology..

[15] Lombardi C, Provini F, Vetrugno R, Plazzi G, Lugaresi E, Montagna P (2003). Pelvic movements as rhythmic motor manifestation associated with restless legs syndrome. Mov Disord.

[16] Baiardi S, La Morgia C, Mondini S, Cirignotta F. A restless abdomen and propriospinal myoclonus like at sleep onset: an unusual overlap syndrome. BMJ Case Rep. 2015;2015. 10.1136/bcr-2014-206679. 10.1136/bcr-2014-206679 PMC4386442 25820108

[17] Pengo MF, Won CH, Bourjeily G (2018). Sleep in women across the life span. Chest..

[18] Manconi M, Ulfberg J, Berger K, Ghorayeb I, Wesstrom J, Fulda S (2012). When gender matters: restless legs syndrome. Report of the "RLS and woman" workshop endorsed by the European RLS Study Group. Sleep Med Rev.

[19] Wolkove N, Elkholy O, Baltzan M, Palayew M (2007). Sleep and aging: 2. Management of sleep disorders in older people. CMAJ.

[20] Turrini A, Raggi A, Calandra-Buonaura G, Martinelli P, Ferri R, Provini F (2018). Not only limbs in atypical restless legs syndrome. Sleep Med Rev.

[21] Valko PO, Siccoli MM, Bassetti CL (2009). Unilateral RLS with predominantly ipsilateral PLMS and variable response to dopaminergic drugs: a variant of idiopathic RLS?. Eur J Neurol.

